# Cesarean scar pregnancy with molar pregnancy: A case report and literature review

**DOI:** 10.1097/MD.0000000000044140

**Published:** 2025-09-05

**Authors:** Yuanmei Deng, Ya chai Li, Meng Li, Liwei Yan, Jiarui Mi

**Affiliations:** a Department of Obstetrics and Gynecology, Hebei University Affiliated Hospital, Hebei, China.

**Keywords:** cesarean scar pregnancy, diagnosis, molar pregnancy, rare complication, treatment

## Abstract

**Rationale:**

Cesarean scar pregnancy with molar pregnancy is a rare but high-risk pregnancy complication characterized by the implantation of a fertilized egg in the uterine scar following cesarean section, accompanied by pathological manifestations of a hydatidiform mole. This paper reports a clinical case of hydatidiform mole in a cesarean scar and reviews the literature to understand its diagnosis and treatment strategies.

**Patient concerns:**

We reported a 33-year-old woman who presented to our hospital with intermittent vaginal bleeding for over 2 months following uterine curettage.

**Diagnoses:**

Transvaginal ultrasonography, serum human chorionic gonadotropin, pelvic magnetic resonance imaging, and histological examination confirmed the disease.

**Interventions:**

The patient underwent bilateral uterine arterial embolization, suction evacuation, and transabdominal surgery.

**Outcomes:**

Postoperative histological examination of the tissue revealed hydatidiform moles.

**Lessons:**

Molar pregnancy in the cesarean scar is difficult to differentiate from normal cesarean scar pregnancy with serum human chorionic gonadotropin, sonogram, or magnetic resonance imaging. This case demonstrates the diagnosis and treatment strategy for cesarean scar pregnancy with molar pregnancy.

## 1. Introduction

Cesarean scar pregnancy with molar pregnancy is an extremely rare complication. Although its incidence is low, it carries significant clinical risk. Cesarean scar pregnancy (CSP) refers to the implantation of a fertilized egg in the scar of a previous cesarean section, whereas molar pregnancy is an abnormal pregnancy characterized by the abnormal proliferation of trophoblasts and interstitial edema. When these 2 conditions occur simultaneously, a molar pregnancy in the cesarean section scar is formed. This situation is very rare, with a complex pathological mechanism, and is prone to serious complications, such as massive bleeding and uterine rupture, posing a significant threat to the patient’s life and health.

In recent years, with the increase in cesarean section rates and advancements in diagnostic technology, reports of cesarean scar pregnancies have also gradually increased. However, cases of hydatidiform moles in cesarean section scars remain extremely rare. Owing to the lack of large-scale clinical research, there is still considerable controversy regarding the diagnostic criteria and treatment protocols for molar pregnancies in cesarean scars. Therefore, this paper aims to discuss the clinical features, diagnostic methods, and treatment progress of a molar pregnancy in a cesarean scar by reporting and analyzing the case, combined with a review of relevant domestic and international literature, to provide a reference for clinicians and improve their understanding, diagnosis, and treatment of the disease.

## 2. Case report

A 33-year-old married woman, Li, was admitted to our hospital on October 18, 2024, with intermittent vaginal bleeding for over 2 months following uterine curettage. Her menstrual history: Regular cycles (7/30 days), moderate flow, no dysmenorrhea. Obstetric history: Gravida 7, Para 3. Cesarean deliveries (2010, 2014, 2017). Induced abortions ×4.

Two months prior: Transvaginal ultrasound (TVUS) at local hospital revealed heterogeneous intrauterine echoes and elevated serum β-human chorionic gonadotropin (hCG; approx. thousands mIU/mL, exact value unknown). Diagnosed as incomplete abortion; underwent dilation and curettage (D&C). Pathological examination of tissue was refused by the patient.

Post-D&C course: Intermittent scant vaginal bleeding for >2 months with no resumption of regular menses. No abdominal pain, and no follow-up TVUS/β-hCG testing.

**Figure 1.**
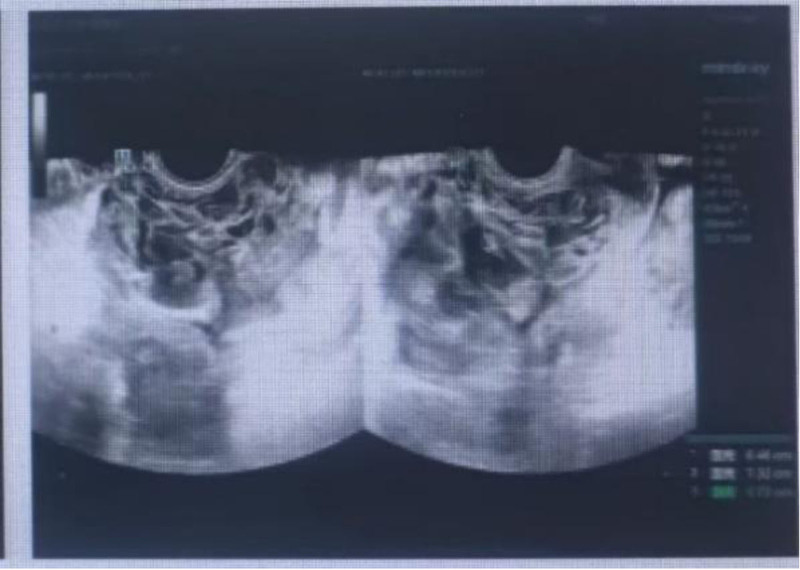
Ultrasound image showing a cluttered echo mass in the uterine cavity with fine, punctate cysts.

One day prior to admission: Increased vaginal bleeding. TVUS at our hospital: Complex mixed-echo mass (12.2 cm × 7.8 cm × 5 cm) in uterine cavity with cystic components (Fig. 1). Myometrial thinning (minimal thickness: 0.5 cm). Serum β-hCG: 1,15,824.00 mIU/mL. Gynecological examination: Cervix: Dark-red blood flow (menstrual-volume equivalent), no cyanotic nodules. Uterus: Enlarged to 16-week gestational size, non-tender. Adnexal: Unremarkable. Initial diagnosis: Retained products of conception with hematoma. CSP? Gestational trophoblastic disease? High risk of massive hemorrhage → Emergency bilateral uterine artery embolization (UAE) performed prior to repeat D&C.

**Figure 2.**
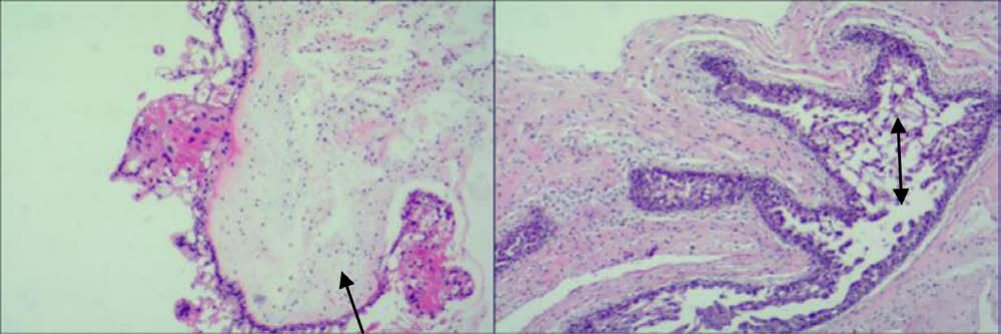
Necrotic tissue, villous interstitial edema (↑) with moderate proliferation of nourishing cells (↕).

Post-UAE day 3: Ultrasound-guided D&C. Evacuated material: Mostly blood clots mixed with decidual tissue and focal hydropic vesicles. Pathology report (Fig. 2): Decidual tissue with hydropic villi, stromal edema, and moderate trophoblastic cell proliferation. Final Diagnosis: Complete hydatidiform mole.

**Figure 3.**
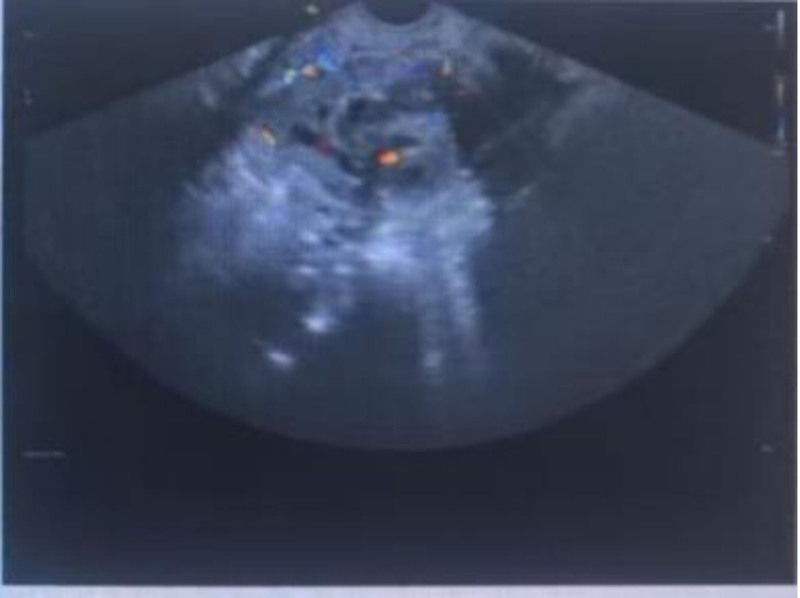
Transvaginal color ultrasound revealing a cluttered echo mass in the lower uterus with abundant blood flow signals.

**Figure 4.**
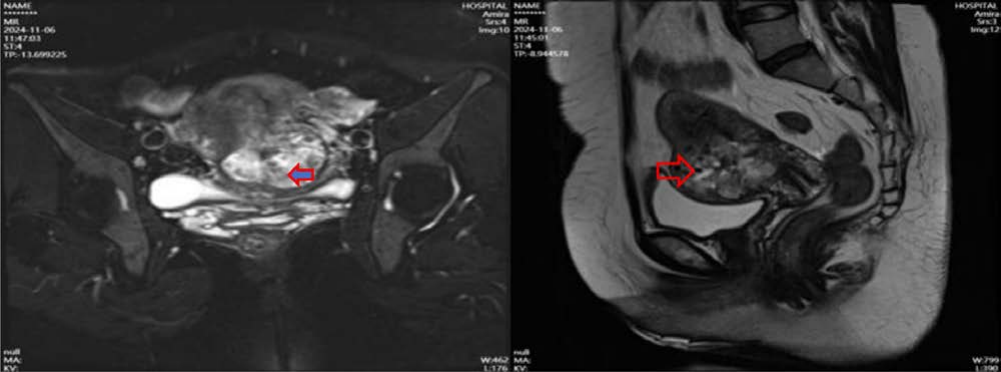
Pelvic MRI showing a mass in the lower anterior uterine wall protruding with a thin muscular layer. MRI = magnetic resonance imaging.

**Figure 5.**
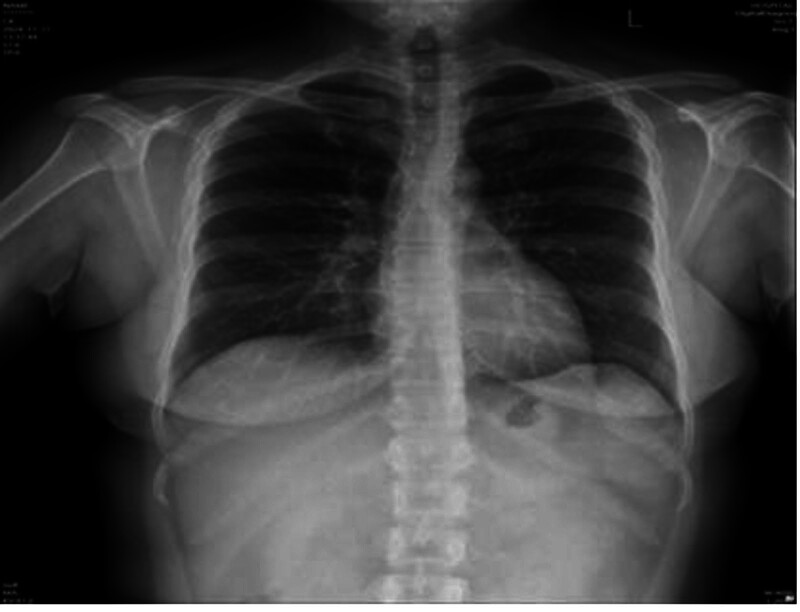
Chest x-ray no significant abnormalities.

**Figure 6.**
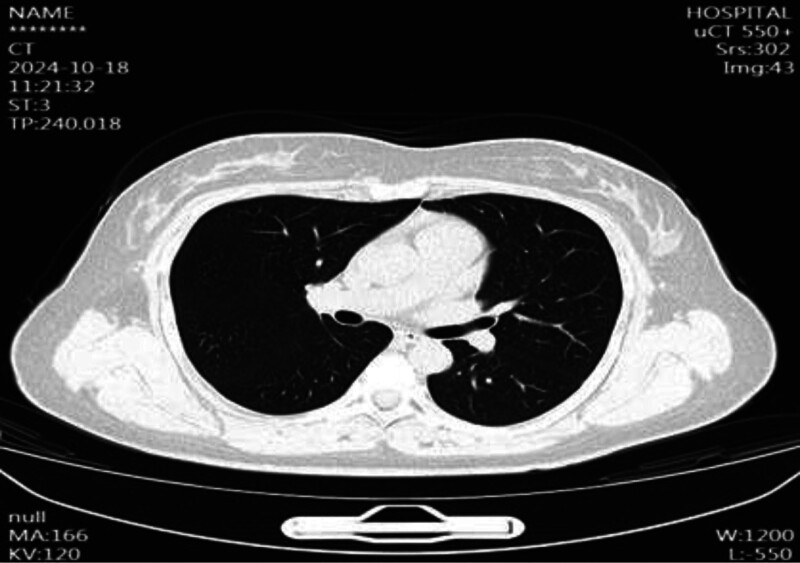
CT showing no significant abnormalities. CT = computed tomography.

**Figure 7.**
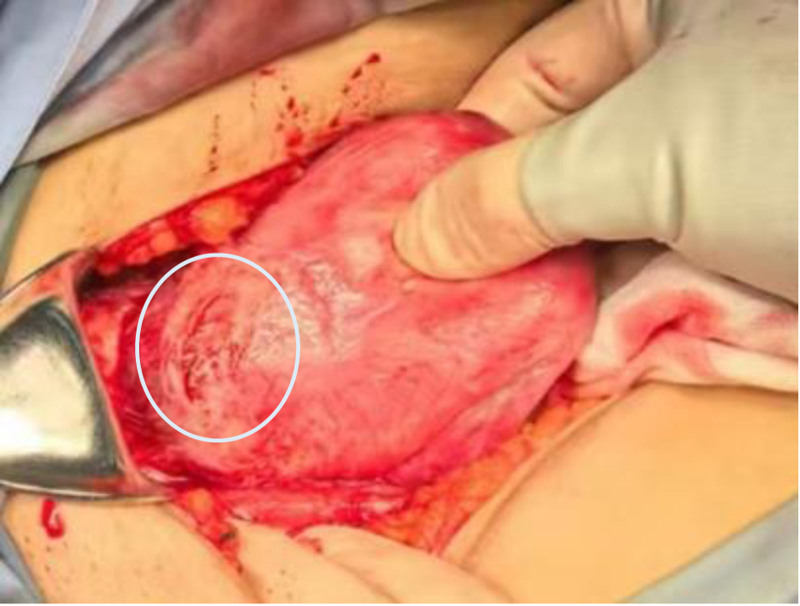
Intraoperative exploration revealing a purplish-blue lesion protruding from the lower uterine segment.

Postoperative β-hCG Monitoring: Day 7: 15,066 IU/mL, day 15: 14,921 IU/mL, day 22: Rebound to 15,711 IU/mL → Suspect persistent trophoblastic disease. Imaging Follow-up: TVUS (Day 22): Heterogeneous mass (5.7 cm × 5.0 cm × 4.1 cm) at lower uterine segment with rich vascular flow on CDFI (Fig. 3). Pelvic magnetic resonance imaging (MRI): 5.4 cm × 4.3 cm mass at anterior uterine wall, exophytic growth, myometrial thinning (Figs. 4 and 5). Chest x-ray/CT: No metastasis (Figs. 6 and 7).

**Figure 8.**
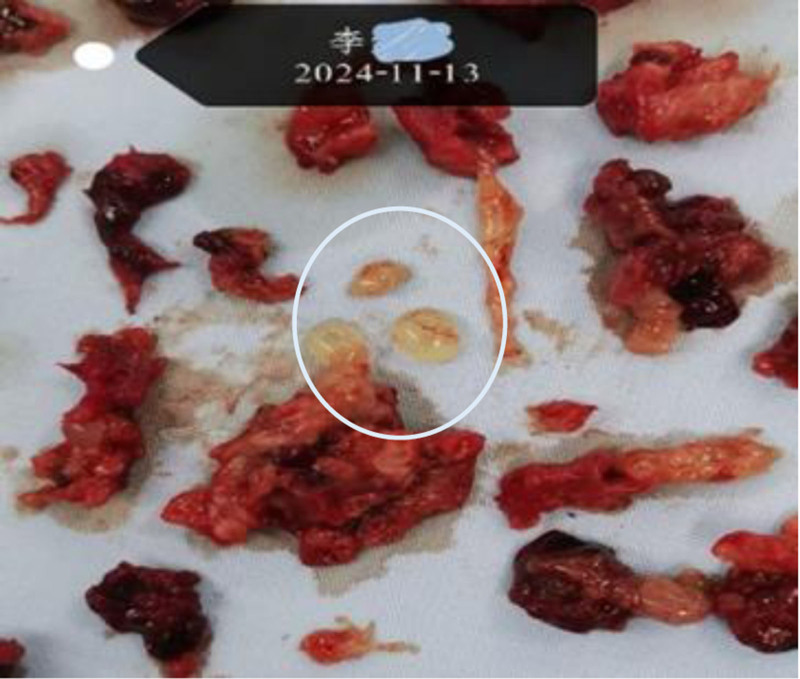
Gross examination of the removed gestational tissue showing vesicular tissue.

**Figure 9.**
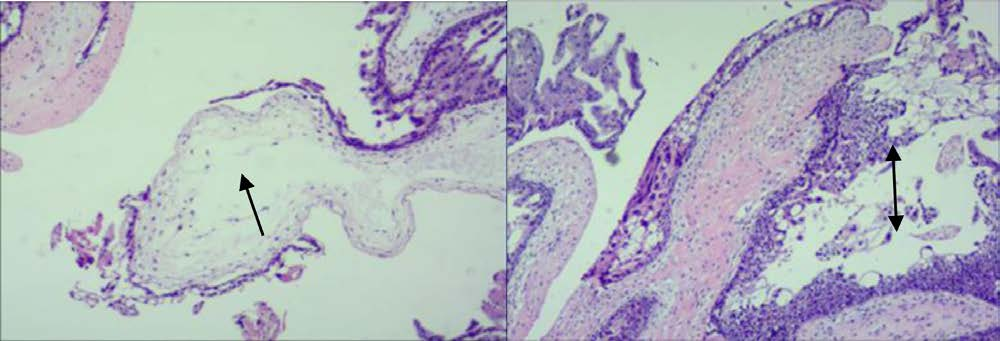
Villous interstitial edema (↑) with moderate proliferation of nourishing cells (↕), consistent with hydatidiform mole.

Lesion classified as Type III CSP (mass predominantly bulging toward serosa; unfit for hysteroscopic resection). Underwent abdominal mass excision + uterine repair on November 13, 2024. Intraoperative findings: Bluish-purple mass (5 cm × 5 cm) at uterine scar site with thin serosal coverage and vascular congestion (Fig. 6). Hydropic vesicles confirmed (Fig. 8). Pathology (Fig. 9): Hydatidiform mole, hydropic villi, and moderate trophoblastic hyperplasia. Smooth muscle and adipose tissue adherent.

**Figure 10.**
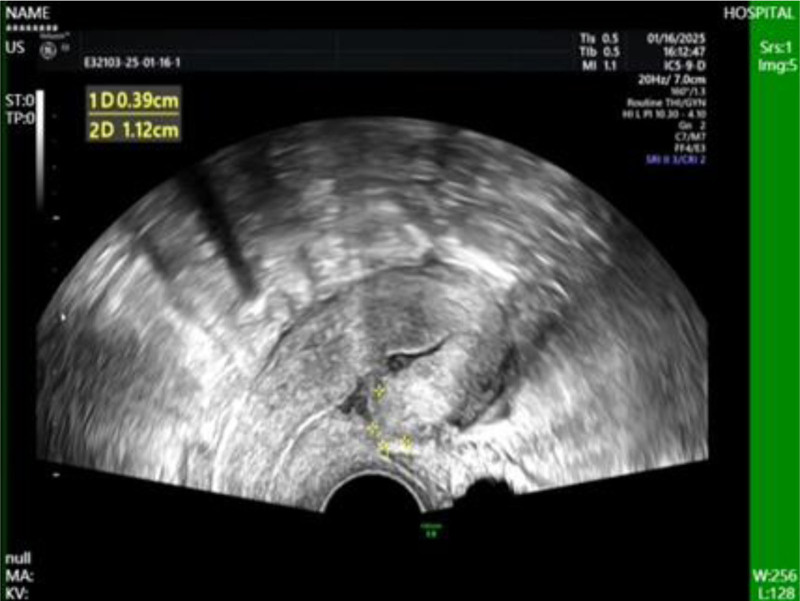
Postoperative transvaginal ultrasound showing a localized hypoechoic area.

**Figure 11.**
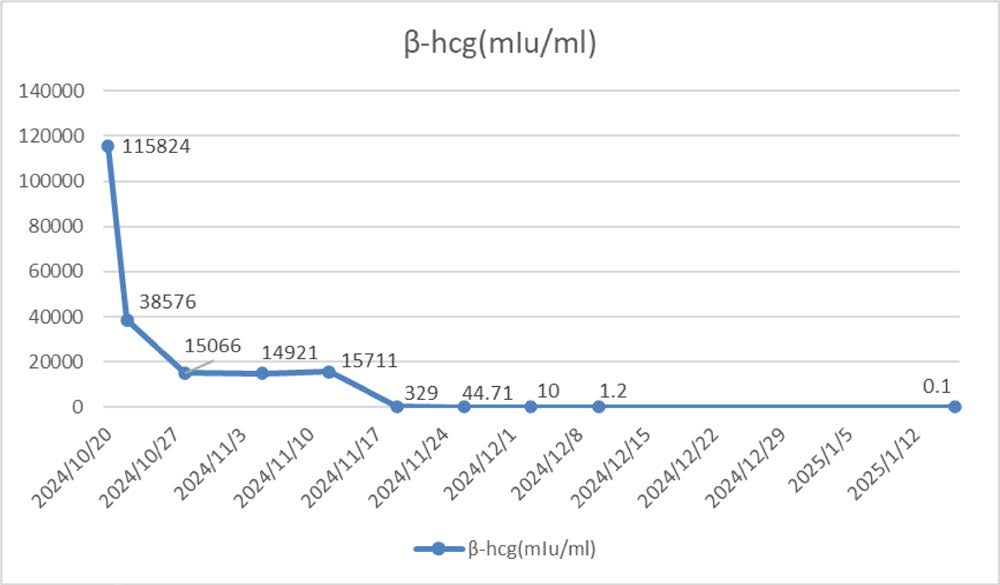
Changes of β-hCG levels in the blood. HCG = human chorionic gonadotropin.

Day 13 TVUS: Minimal fluid collection (1.3 cm × 0.9 cm) at scar site; no abnormal vascularity (Fig. 10). β-hCG declined progressively to normal range by day 27 (Fig. 11). Regular menses resumed, no abnormal bleeding. The flowchart is as follows.

Final Diagnosis: Complete Hydatidiform Mole within Cesarean Section Scar. Follow-up recommendations: Strict contraception (nonhormonal methods preferred). Continued β-hCG surveillance for 12 months to exclude malignant transformation.

## 3. Discussion

The pathogenesis of cesarean scar pregnancies remains unclear. Current research suggests that it may be related to delayed development of the fertilized egg, defective healing of the uterine scar, and abnormal development of the uterine decidua.^[1]^ Defects in the myometrium and vascular hyperplasia at the site of the uterine scar may form the pathological basis of CSP.^[2,3]^ Diagnosis and classification primarily rely on ultrasound findings.^[4]^ Once diagnosed, pregnancy termination must be performed as early as possible.^[5]^ Treatment methods include ultrasound-guided or hysteroscopic curettage, laparoscopic or open surgical removal of the gestational lesion, among others.^[6,7]^ Due to the thin myometrium at the uterine scar site and poor contractility, severe complications, such as massive hemorrhage or hemorrhagic shock, may occur during treatment. Therefore, for patients at a high risk of bleeding, UAE may be considered preoperatively to reduce the risk of significant bleeding.

The occurrence of hydatidiform moles is often associated with various factors such as cytogenetic abnormalities, viral infections, and endocrine disorders. Its clinical manifestations include a uterus significantly larger than expected for the corresponding gestational age (occurrence rate 28%), vomiting (occurrence rate 8%), abnormally elevated HCG levels, ovarian theca lutein cysts (occurrence rate 15%), and hypertension in early to mid-pregnancy (occurrence rate 1%).^[8]^ Ultrasound examinations typically reveal an intrauterine cavity without a discernible gestational sac filled with anechoic areas of varying sizes, presenting a honeycomb-like appearance, also known as the “snowstorm sign.” The 2024 Chinese Expert Consensus on the Diagnosis and Treatment of Hydatidiform Mole recommends that the clinical diagnosis of hydatidiform mole be based on clinical manifestations, physical signs, ultrasound findings, and serum β-hCG levels, with pathology being the “gold standard” for diagnosis. Upon confirmation of hydatidiform mole, D&C should be performed as soon as possible to terminate pregnancy. During treatment, vigilance against massive hemorrhage is necessary. After treatment, close monitoring of HCG levels is essential. If the decline in HCG is unsatisfactory or if it rises again after a decline or plateau, the occurrence of gestational trophoblastic neoplasia should be suspected. However, before making such a diagnosis, retention of pregnancy products must be ruled out.

Cesarean scar pregnancy with molar pregnancy, as the name suggests, is a rare condition in which molar tissue implants and abnormally proliferates at the site of a previous uterine scar, forming a special type of ectopic pregnancy and trophoblastic disease. Although its incidence is extremely low, it is associated with significant risks. Currently, there are no definitive statistical data available, with only isolated case reports documented.^[1,3]^ The clinical presentation, diagnosis, and treatment require an individualized approach that considers the characteristics of both scar pregnancy and hydatidiform moles.

Owing to the rarity of gestational trophoblastic disease at the scar site in clinical practice, there is currently no unified standard for its diagnosis and treatment, with information limited to case reports. Isha Kriplani^[9]^ reviewed the literature and found only 8 reported cases of cesarean scar hydatidiform mole pregnancies. All patients underwent at least 2 curettage procedures, with abnormally elevated β-hCG levels. Clinical manifestations vary widely, with the most common symptom being vaginal bleeding lasting more than 1 month. Postoperative specimens should be sent for histological examination^[9,10]^ The diagnosis of gestational trophoblastic disease in scar areas is mainly based on clinical manifestations, HCG changes, imaging examinations, and postoperative pathology. Ultrasound plays an important role in the diagnosis of this disease, but still has certain limitations. Magnetic resonance imaging can be used as a necessary supplement to better understand the relationship between pregnant tissue and scars, as well as blood flow, or the possible presence of cystic signals resembling molar pregnancy.^[10]^ However, for extremely early or atypical scar site trophoblastic diseases, both ultrasound and MRI lack specificity. Yamada and Ohira^[7]^ reported that only 1 case of ectopic molar pregnancy could be diagnosed with MRI before surgery in 31 cases.

Due to the thin myometrium at the uterine scar site, poor contractility, and the influence of the scar, blood sinuses are difficult to close. Moreover, molar tissue is more invasive than normal pregnancy tissue, making it highly prone to uncontrollable massive hemorrhage. Wang Meng^[11]^ and others chose ultrasound-guided curettage under the condition of having blood preparation and rescue measures in place. To prevent risks such as incomplete evacuation and chorionic implantation, interventional chemoembolization of the uterine artery was performed after curettage. Some scholars believe that preoperative chemotherapy should be administered first, and after the blood HCG level returns to normal or near normal, laparoscopic uterine scar lesion removal surgery can be performed to reduce intraoperative bleeding and trophoblastic cell transfer. This approach allows for the removal of the lesion while suturing and repairing the uterine scar, correcting the defect at the scar site, reducing the adverse effects of lesion erosion, preserving the uterus and fertility function, and resulting in a good prognosis.^[8,12,13]^ Literature has also explored the value and efficacy of hysteroscopy in the treatment of hydatidiform mole and has given it recognition.^[14]^ Shi Liping^[1]^ and others chose hysteroscopic curettage and secondary curettage after interventional embolization of the uterine artery, followed by regular ultrasound and blood HCG reviews until normalization. Nian Chun et al’^[15]^ suggested that reducing the pressure of the distension medium can to some extent decrease the probability of distension fluid spilling into the abdominal cavity and implantation of malignant tumor cells. Thus, hysteroscopy does not artificially cause the transfer of trophoblastic cells.

In this case, preoperative ultrasound at the local hospital did not indicate a close relationship between the gestational tissue and the uterine scar site. Consequently, curettage was performed following the general method for terminating pregnancy, and no pathological examination was conducted. This led to a missed opportunity for the first diagnosis of trophoblastic disease and an underestimation of the subsequent risk of bleeding or residual gestational tissue, resulting in reduced awareness of the need for follow-up. Postoperative vaginal bleeding persisted for >2 months. A repeat ultrasound examination revealed a large amount of accumulated blood within the uterine cavity, causing the uterus to enlarge to the size of a 4+ month pregnancy. However, ultrasound could not clearly define the relationship between the residual tissue and the scar, and the blood HCG levels were abnormally elevated. We suspected gestational trophoblastic disease, but the patient was at imminent risk of massive hemorrhage. The immediate priority was to evacuate the intrauterine tissue as soon as possible under precautions to prevent bleeding and obtain pathological results to confirm the diagnosis. Therefore, curettage was performed after UAE, and the postoperative pathology was consistent with the presentation of a hydatidiform mole, confirming the diagnosis of trophoblastic disease. However, it remains unknown whether there is scar pregnancy. Thus, we instructed the patient to closely follow up with ultrasound monitoring of the intrauterine situation and to determine whether the HCG levels were satisfactorily decreased. Unfortunately, ultrasound on postoperative day 13 indicated a heterogeneous mass at the scar site, and although the HCG levels had significantly decreased, there was a trend of increase again (1,15,824–15,066–15,711) mIU/mL. To understand the condition at the scar site, a pelvic MRI was conducted, which suggested chaotic echoes in the lower segment of the uterus protruding towards the serosal layer, not excluding the possibility of residual trophoblastic cells locally after the removal of the hydatidiform mole tissue, nor completely ruled out the possibility of invasive mole tissue infiltrating from the uterine cavity into the myometrium. To thoroughly remove the lesion and repair the uterine scar, transabdominal removal of the gestational tissue at the uterine scar site and uterine repair were chosen during the second hospitalization, during which vesicular tissue was again observed, which also provided support, preserving the patient’s uterus and fertility, and improving her future quality of life. To determine whether to proceed with prophylactic chemotherapy postoperatively, the 2024 Chinese Expert Consensus on the Diagnosis and Treatment of Hydatidiform Mole recommends prophylactic chemotherapy only for patients with complete hydatidiform mole who have difficulty in follow-up or have high-risk factors, including: age > 40 years; HCG > 5,00,000 U/L; uterus significantly larger than the corresponding gestational age; ovarian theca lutein cyst diameter >6 cm; and history of repeated hydatidiform moles. The risk of local invasion and/or distant metastasis is as high as 37% for those over 40 years of age and up to 56% in those over 50 years of age. This patient did not have these high-risk factors, so postoperative follow-up was strictly conducted according to the requirements for hydatidiform mole and subsequent family planning management. Currently, HCG values are normal for 3 consecutive tests, and menstruation has returned to normal twice. The diagnosis and treatment process of this patient was tortuous and complex, considering the characteristics and treatment principles of both uterine scar pregnancy and hydatidiform mole, and the patient ultimately achieved a good outcome.

Throughout the treatment of this case, we have engaged in reflection and identified several areas for improvement: First, should the patient undergo an MRI examination during their first visit to our hospital to clarify the relationship between residual lesions and scars, as well as whether there are signs of molar pregnancy? during the first curettage in our hospital, after removing the hematoma, if further hysteroscopy is performed to remove visible lesions as much as possible under hysteroscopy, is it possible to avoid a second open surgery. It is our hope that through the reporting of this case, we can enhance the clinical experience, broaden our perspectives, and recognize the possibility of hydatidiform mole occurring at the site of a cesarean scar. We aimed to provide patients with a precise diagnosis and treatment, minimize detours, and improve the standard of clinical diagnosis and treatment.

## Author contributions

**Conceptualization:** Yuanmei Deng, Ya chai Li.

**Data curation:** Yuanmei Deng, Ya chai Li.

**Data collection:** Yuanmei Deng, Ya chai Li, Meng Li, Liwei Yan, Jiarui Mi.

**Formal analysis:** Yuanmei Deng, Ya chai Li.

**Investigation:** Meng Li, Liwei Yan.

**Methodology**: Jiarui Mi.

**Project administration**: Jiarui Mi.

**Project development:** Yuanmei Deng, Ya chai Li.

**Supervision**: Jiarui Mi.

**Writing – original draft:** Yuanmei Deng, Ya chai Li.

**Writing – review & editing**: Yuanmei Deng, Ya chai Li.
